# Meningovertebral ligaments could be a barrier for migration of a herniated intervertebral disc: An anatomical study

**DOI:** 10.3389/fsurg.2022.969244

**Published:** 2022-09-07

**Authors:** Kamil Krystkiewicz, Mateusz Maślanka, Tymon Skadorwa, Bogdan Ciszek, Marcin Tosik, Jacek Furtak

**Affiliations:** ^1^ Department of Neurosurgery and Neurooncology, Copernicus Memorial Hospital, Łódź, Poland; ^2^ Department of Descriptive and Clinical Anatomy, Medical University of Warsaw, Warsaw, Poland; ^3^ Department of Pediatric Neurosurgery, Bogdanowicz Memorial Hospital for Children, Warsaw, Poland; ^4^ Department of Neurosurgery, 10th Military Research Hospital, Bydgoszcz, Poland

**Keywords:** disc herniation, meningovertebral ligaments, anterior epidural space, intervertebral disc, sciatica, meningovertebral ligaments in disc herniation

## Abstract

**Purpose:**

Intervertebral disc degeneration can manifest as sequestration. In most cases, the material could be found ipsilateral to the annular tear; however, a contralateral migration is also possible. We present an anatomical description of anterior meningovertebral ligaments (MVLs) as a possible barrier for disc migration.

**Methods:**

Anatomical dissection of 20 fresh human cadavers was carried out. Complete lumbar laminectomies with facetectomies were performed. All lumbar segments were exposed. Morphologic and morphometric descriptions of anterior MVLs were presented, with special attention to possible routes of herniated disc migration.

**Results:**

Anterior MVLs were present in all cases. They were divided in three separate groups: medial, lateral, and attached to the nerve roots. The medial group was the thickest, its mean length was 26.2 ± 1.2 mm, and it had no attachment to the disc in 51% of cases. The lateral group was less firm than the medial group, its mean length was 26.9 ± 1.0 mm, and it had no relation with the disc in 47% of cases. Ligaments related to the nerve root were the most delicate and always attached to the intervertebral disc. Their mean length was 14.9 ± 1.8 mm.

**Conclusions:**

The medial group of anterior MVLs are strong connective tissue bands dividing the anterior epidural space. The lateral group is more delicate, and in most cases, lateral MVLs lack annular attachment. MVLs could be an anatomical barrier for disc migration in particular cases.

## Introduction

Intervertebral disc degeneration can manifest as herniation, meaning that a portion of the nucleus pulposus pushes through the annulus into the spinal canal. There are several morphologic variants of disc herniation, one of which is sequestration. It is defined as a displacement of the nucleus material, which loses continuity with the disc. It is a common manifestation of this disease and is diagnosed even in 47% of cases of disc herniation (1). The main clinical presentation is a sciatica—a pain radiating along the dermatome corresponding to the compressed spinal nerve. The disc material is known to migrate in the vertebral canal caudally, rostrally, posteriorly to the dural sac, or intradurally (2, 3). Typically, the fragment is found ipsilaterally to the annulus tear. However, rare documented cases of contralateral migration of the lumbar disc herniation have been published (4). Several vertebral canal structures are described, which may block a free disc movement from side to side: the epidural fat, venous plexus, posterior longitudinal ligament (PLL), and meningovertebral ligaments (MVLs), the so-called Hofmann ligaments (5). MVLs are divided into three separate groups based on the topography of attachments: anterior, lateral, and posterior ligaments (5). Anterior ones are connective tissue bands binding the ventral surface of the dura with the vertebral canal structures. Lateral ligaments are located in the coronal plane, between the anterior and the posterior ones. Posterior MVLs could be found on the dorsal surface of the dural sac. Anterior MVLs were previously described by Hofmann (6), so an eponymic description—Hofmann’s ligaments could be found in the literature (2, 7). Anterior MVLs could be further divided into three separate groups: medial (bundles attached to the anterior part of the dura and to the PLL), lateral (connecting the anterolateral surface of the dural sac and the lateral aspects of the PLL), and radicular (from the dural sleeve of the proximal segment of the spinal nerve to the ipsilateral pedicle). The first two groups were described originally by Hofmann. However, he was not aware of the radicular ones, which were described in further studies (5). It seems that the main role of MVLs is support and stabilization of the dural sac during the movement of the spine. It is described that MVLs could limit the stretching of the spinal nerve when large, anteriorly located disc materials displace the spinal nerves posteriorly. There are some descriptions of MVLs as a contributing mechanism in the pathophysiology of the sciatica in the same mechanism (8, 9). However, anatomical data on the topographic relations of anterior MVLs with regard to their role as a barrier for herniated disc migration are limited.

## Materials and methods

### Specimens

Cadavers were collected from a voluntary donation program and were a part of the collection of the Department of Clinical and Descriptive Anatomy, Medical University of Warsaw, Poland. The research was conducted in accordance with the Polish Death and Funeral Act and with the relevant guidelines and regulations. The Institutional Ethics Committee was informed about the ongoing study, and it stated that there was no need for obtaining its approval.

Twenty fresh cadaveric spine specimens (10 males and 10 females) were used in the study. The estimated age of the specimens ranged from 30 to 70 years. None of them presented either neoplastic diseases or an infection of the vertebral column or paravertebral tissues. None of the specimens had scars on the back. There was no past medical history of anterior spinal procedures and instrumentation.

### Technique

The skin was incised longitudinally from the level of S1 to the level of the T12 spinous process. Paravertebral muscles were dissected and retracted in a standard fashion. Complete laminectomies with facetectomies along the lumbar spine were performed. The spinal dura was exposed. Then, each spinal nerve was followed to its own intervertebral foramen and elevated ventrally. The membranous tissue was evaluated and each spinal nerve was cut and retracted posteriorly. To prevent a falsification of the measurements caused by an elevation of the dura, we did not measure the anterior–posterior dimension of the ligaments. The cranio-caudal length of the MVLs was more important for our study, as it was not distorted by the elevation maneuver. The MVLs were carefully dissected from the epidural fat. The ligaments were described morphologically and documented photographically. Topographic relations to the spinal structures were evaluated. A tissue resistance during blunt dissection was assessed in the context of intervertebral disc herniation.

### Statistical analysis

The statistical analysis was performed by using StatSoft Statistica software (version 13 PL) and Microsoft Excel 2007. Continuous variables were described using means and standard deviations, and categorical variables were summarized using frequencies. A *p*-value <.05 was considered statistically significant.

## Results

### Ligaments

**Figure 1:**
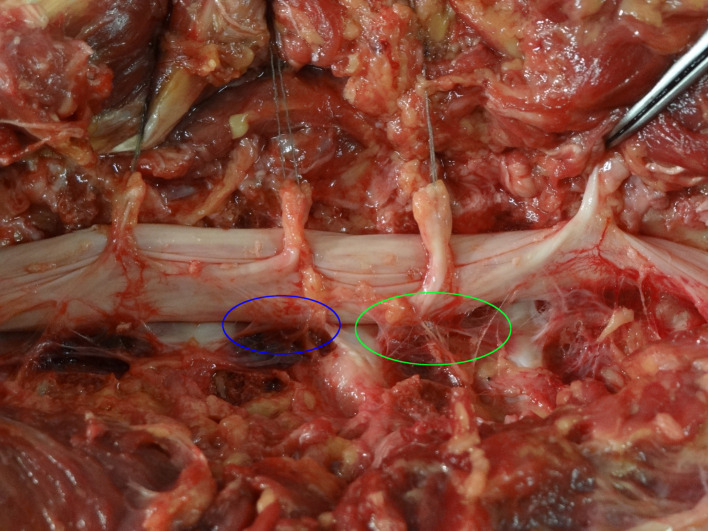
Presentation of the medial (blue) and lateral (green) meningovertebral ligaments. Spinal nerves are transected and elevated for better presentation purposes.

**Figure 2:**
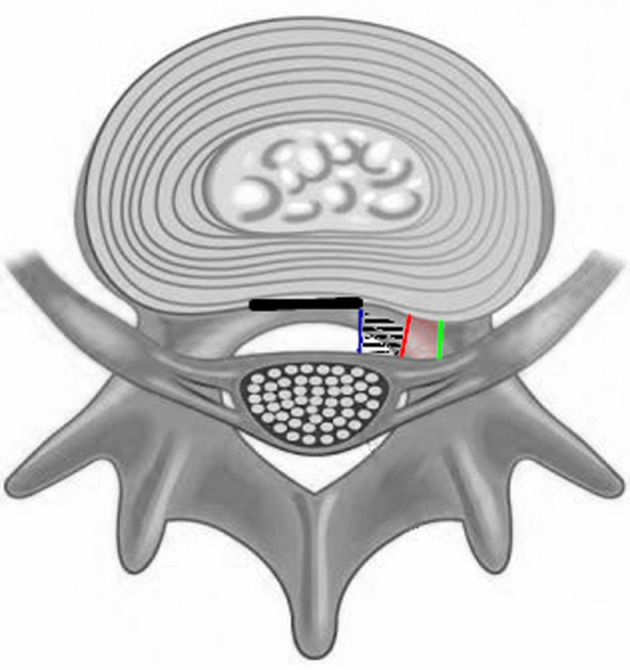
Schematic drawing presenting location and attachments of the ligaments on the axial cut of the spinal canal. Thick dark line—posterior longitudinal ligament, blue line—medial MVL, red line—lateral MVL, green line—radicular MVL, black dashed area—medial part of the anterior epidural space, red area—lateral part of the anterior epidural space.

Anterior MVLs were observed in each of the 20 cases (40 sides) dissected. There were no statistically significant differences between sides (*p* = 0.34) and sexes (*p* = 0.84). The ligaments were visible only when the dura was elevated posteriorly (Figure 1). Fat tissue and a venous complex were noted between them. The ligaments were divided into three groups based on the topography of attachment and the relation to the midline (Figure 2). The first group included medial bands attached to the superficial part of the PLL and the anterior surface of the dural sac. The fibers of the ligaments were always directed lateral to the midline, attaching to the lateral margin of the PLL. They were firm, thick, and resistant to blunt dissection. With regard to all lumbar levels, in 51% of cases, there was no attachment to the annulus (Table 1). The attachments to the PLL were observed exclusively at the level of vertebral bodies. However, in the remaining cases, when the fibers were connected with the intervertebral disc, an attachment could be noted only at the endplate level, leaving the annulus surface uncovered.

**Table 1:** Frequency of MVL attachments to the intervertebral disc.

Level	Left			Right		
	Medial	Lateral	Radicular	Medial	Lateral	Radicular
L1–L2	5 (25%)	6 (30%)	20 (100%)	4 (20%)	6 (30%)	20 (100%)
L2–L3	6 (30%)	4 (20%)	20 (100%)	5 (25%)	5 (25%)	20 (100%)
L3–L4	12 (60%)	10 (50%)	20 (100%)	13 (65%)	11 (55%)	20 (100%)
L4–L5	13 (65%)	12 (60%)	20 (100%)	13 (65%)	13 (65%)	20 (100%)
L5–S1	15 (75%)	15 (75%)	20 (100%)	15 (75%)	16 (80%)	20 (100%)

The second group included lateral MVLs, which were located more laterally to the medial group. They connected the anterolateral surface of the dural sac with the posterior surface of the vertebral body (Figures 1, 2). Some fibers were projecting medially to the lateral part of the PLL. In general, they were less resistant than the medial bands. In 5% of cases, the lateral bands were macroscopically similar to the medial ones. In the remaining cases, macroscopic evaluation revealed a less firm structure. In 47% of cases, the lateral group had no attachment to the intervertebral disc, nor to the annulus or the endplate of the vertebra (Table 1).

**Figure 3:**
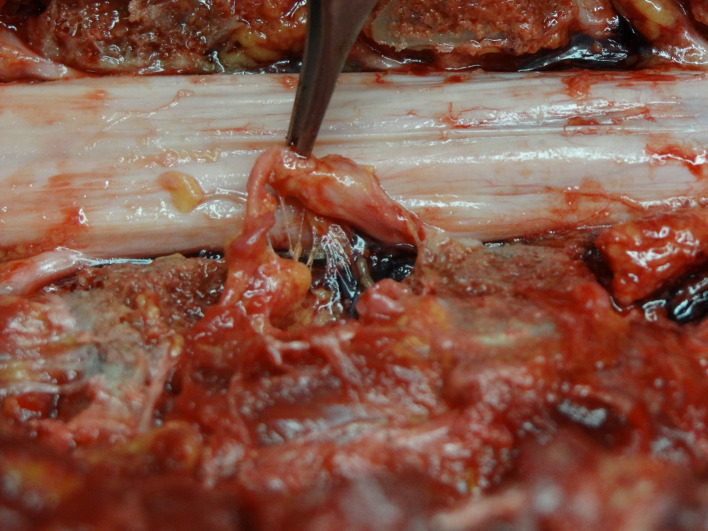
MVLs attached to the spinal nerve *—pedicles, which were removed for the visualization of the intervertebral foramen purposes.

The third topographical group was radicular ligaments. They connected the spinal root with the surrounding spinal bone structures. These thin ligamentous, membrane-like tissues connected the dural sleeves of the spinal nerves with vertebral bodies, ipsilateral pedicles, and the intervertebral disc at the level of the foramen (Figure 3). They were always thin, very delicate, and easily detached after blunt manipulation with the Penfield dissector. An attachment to the intervertebral disc was always present (Table 1). The direction of these ligamentous bands was parallel to the nerve roots. They projected inferiorly and laterally from the surface of the dural sac. Between the sagittally oriented lateral MVL and the obliquely projecting spinal nerves, the anterolateral portion of the epidural space was found (Figure 2). In contrast to the medial ones, it was in the shape of a triangle and limited cranially and caudally by the nerve roots and their MVLs.

The general observation was that the medial and lateral bands were becoming more firm and thick at the lower lumbar levels. It was also noted that the lower the spine level, the greater the length of the ligaments (Table 2). The ligaments most resistant to manipulation and the thickest were the medial ones at the L5–S1 level. The attachments to the discs were more frequent the lower the level of the spine was. However, at L4–L5 and L5–S1, the medial bands had attachments to the intervertebral disc in 64% and 76% of cases, respectively. In all cases, the disc was covered only partially, leaving a large part of the annulus unprotected.

**Table 2:** Measurements of the MVL length with regard to the spine segment.

Level	Left (mm)			Right (mm)		
	Medial	Lateral	Radicular	Medial	Lateral	Radicular
L1–L2	25.0	26.5	13.0	24.5	26.5	13.0
L2–L3	26.0	26.0	13.5	25.0	25.5	13.0
L3–L4	25.5	26.5	15.0	26.0	26.5	14.5
L4–L5	27.5	27.5	16.0	26.5	27.0	16.0
L5–S1	28.0	28.5	17.5	28.0	29.0	28.0

### Anterior portion of the epidural space

Anteriorly to the dural sac and posteriorly to the PLL, the anterior part of the epidural space could be found. It is an anatomical space filled with fat, vessels—mostly venous plexus, and lymphatic channels.

The medial portion of that epidural space is located between the lateral and the medial MVL. Anteriorly, it is bounded by the PLL and posteriorly by the anterior surface of the dural sac. This area communicates with the contralateral one through small fenestrations within the MVL and free spaces between them at the level of the intervertebral disc. There is no anatomical limitation superiorly and inferiorly, so that part of the epidural space continues from the upper lumbar levels to the level of the sacrum. However, this space may be limited by the degeneration of the intervertebral disc or osteophytes of the endplates, which was noted in our dissected cases.

A lateral part of the anterior epidural space can be found between the sagittally oriented lateral MVL and obliquely projecting spinal nerves. In contrast to the medial portion, the lateral one is triangular and bounded superolaterally and inferomedially by nerve roots and their MVL. Directly laterally, a pedicle could be found. Posteriorly, this space communicates with the lateral and posterior portions of the epidural space. Our study protocol did not allow us to evaluate the ligamentous structures in the posterior and lateral portions of the epidural space.

## Discussion

Several clinical aspects of MVLs have been discussed in the literature. Anesthesiologists have highlighted their role as an obstruction during the placement of the epidural catheter (10). Also, their radiological presentation could be useful when making a differentiation between pyogenic and tuberculous spondylodiscitis (11). Some authors have focused on their possible role in the pathophysiology of sciatica, when disc herniation causes an indirect stretching of the spinal nerve through the radicular segment of MVLs (8, 9). However, we did not find any information about the role and anatomical description of MVLs in the context of herniated disc migration. We attempted to evaluate the morphology and morphometry of anterior MVLs, their relation to the intervertebral disc, and the anatomical conditions of herniation and disc migration.

MVLs can be divided into three groups: anterior, lateral, and posterior (7, 8). In the context of lumbar disc migration, the anterior group plays the main role. These structures are connective tissue bands connecting the anterior surface of the dural sac with various spine elements. They were first described in the late 19th century by Trolard (2) and Hofmann (5). Hofmann described them as narrow connections between the dural sac and the PLL. He distinguished two groups of ligaments: medial and lateral. A more recent description was provided by Wiltse (3) and Scapinelli (6). According to more modern descriptions, we recognize three groups of ligaments, two of them known from Hofmann’s paper and the third—radicular, connecting the dural sleeve around the nerve root with the spinal structures. In our study, we found that these structures were constant and could be found in every case. In general, their superior–inferior dimension is larger on the lower level of the lumbar spine (Table 2). A similar tendency was observed with regard to their resistance for blunt dissection. However, the medial MVLs are the most resistant and they constitute the main barrier for medial migration of the herniated disc. We noted that they were strong septa dividing the anterior portion of the epidural space. When an annular tear appears in the middle anterior epidural space, which is the zone between the medial and the lateral MVL, the herniated disc can migrate superiorly or inferiorly without any anatomical limitations. We observed that the lateral MVL was much thinner than the medial MVL and could be ruptured easily by herniated material. This observation could explain a more common migration pattern involving the disc displacement from the subarticular to the foraminal zone. Radicular MVLs are always very delicate structures and are supposed to be no barrier for the migrating nucleus pulposus. In the literature, there is no information about anatomical conditioning of disc migration. Although there are anatomical and radiologic descriptions of the anterior MVL (2, 4, 5, 7), none of them focuses on the aspect of herniated disc migration. Medial MVLs are dense, strong bands of connective tissue firmly attached both to the dural sac and to the vertebral body. Their structure suggests that they could be a barrier for disc migration. However, a half of intervertebral discs observed in the study were not covered by the ligamentous bands. Most of the disc herniations occurred within the lower part of the lumbar spine—L4/L5, L5/S1. The coverage of the annulus was more frequent at those levels than at others—ligament attachments were found in 75% of cases. Interestingly, such coverage is never complete; there is always a part of the disc unprotected by the MVL. Thus, the disc material can migrate through free spaces to the other side. In addition, when sequestration ruptures the PLL, it can migrate freely within the medial anterior epidural space in the cranial or caudal direction. When an annulus rupture occurs in the subarticular zone, the shortest way for disc migration is the ipsilateral lateral portion of the epidural space. In 95% of cases, lateral MVLs are delicate structures, less firmly attached to spine elements. Moreover, in 90% of cases, they are not attached to the annulus. Probably, they are no barrier for sequestration, similar to radicular MVLs. Hypothetically, we could present a situation in a static model when a contralateral migration may occur. When lateral MVLs were firm and covered most parts of the disc annulus ipsilaterally, they could prevent a lateral migration of the herniated disc. With weak medial MVLs, not covering the annulus, a sequestered disc could migrate to the contralateral direction, because medial MVLs would not provide resistance sufficient enough to prevent migration. Such anatomical conditions would provide a free way for contralateral migration.

The disc material may include the nucleus pulposus, the annulus bundles, the endplate cartilage, or bone fragments. Wiltse (3) reported a widely accepted system of standardizing the nomenclature used in describing the location of the spinal pathologies. According to this system, the intervertebral disc circumference is divided into four zones: central, subarticular, foraminal, and extraforaminal. In each of them, an annular tear may lead to the development of disc herniation. Migration of sequestered material is quite common and can be observed in 35%–72% of cases (7, 9). The disc can migrate superiorly and inferiorly, either under the PLL or in the anterior portion of the epidural space. In the latter case, such a displacement is easier. In our study, we found that there was no anatomical limitation superiorly and inferiorly, so the medial segment of that part of the epidural space continued from the upper lumbar levels to the level of the sacrum. However, this space could be limited by prominent intervertebral disc degeneration or by osteophytes of the endplates, which was also noted in our study. We did not provide an analysis of these degenerative changes in the context of disc migration, due to technical limitations in our study.

The best method to evaluate the degeneration of the intervertebral disc is MRI. Anatomical inspection, when the structure of the vertebral canal is changed, carries a risk of misdiagnosis. A lateral migration means that the nucleus pulposus changes its position from the subarticular to the foraminal zone. It seems to be the most common migration pattern (12). Most of the symptomatic cases manifest clinically as radiculopathy ipsilateral to the annular tear. However, in some cases, a sciatica contralateral to disc herniation can be observed (13). Several mechanisms have been discussed, including hypertrophy of the ligamenta flava, spinal canal stenosis, epidural fat displacement, venous congestion, and displacement of the contralateral nerve roots (14).

A situation where radiculopathy is ipsilateral to the annular tear and disc sequestration, and with the symptoms switching to the contralateral limb after some time, is a unique one. The literature contains very limited data about such cases. Mobbs (4) described the case of a 32-year-old man with a right-sided sciatica. MRI revealed a severe disc herniation at the L2/L3 level and right-sided compression of the L3 nerve. The patient was scheduled for surgery—microdiscectomy at the L2/L3 from the right side. Meanwhile, because of the paraspinal muscle spasm, the symptoms were relieved as a result of some movement of the spine, and after this, the right-sided sciatica remised. However, he developed new symptoms—left-sided L5 radiculopathy. New MRI was performed, revealing a sequestered disc material at the L4/L5 level on the left side and no pathologic mass in the previous location. In addition, no signs of new annular tear were noted. This case presented a very rare medial contralateral migration pattern. Based on our study, where dissected ligaments were more prominent in the lower segments of the lumbar spine, we could hypothesize that such an unusual migration pattern is easier at the higher level of the spine (L2–L3), as in the case presented by Mobbs.

Microdiscectomy is a common spine procedure performed in every neurosurgical department or spine unit. Every surgeon should be aware of the vertebral canal anatomy, especially in the anterior portion of the epidural space, where the herniated disc is usually found. This area is frequently explored for the pieces of the herniated intervertebral disc. Medial MVLs are almost always firm structures, and their attachment to the ventral surface of the dura is strong. In some cases, a blind manipulation of MVLs could be potentially a cause for dural tear (15). What is more, MVLs could be an anatomical barrier for the surgeon when central disc herniation is managed. Lateral and medial MVLs should be transected before the main mass of the herniation could be approached. Apart from the problem of degenerative spine disease, a knowledge about MVLs is crucial when handling oncological cases. During the separative procedure, the anterior surface of the dura is released from the tumor. In some cases, the PLL is intact and the middle and lateral MVLs should be released from the dura before the PLL transection. A similar technique is mandatory during partial or *en bloc* corpectomy. Based on our results, a transection of MVLs should be done at the disc level, where there is a lack of attachments to the annulus. It is easier to begin the cut in the gap between the ligaments than to begin it in the middle of the vertebra.

We attempted to provide a description of MVLs and the epidural space in the context of herniated disc migration. However, the limitation of our study is that it is only an anatomical description. We have not provided any quantitative analyses of the resistance of ligaments and their mechanical properties. What is more, we have presented only a static model, without an inclusion of forces observed in the natural model. Nevertheless, such an anatomical analysis provides additional knowledge for the description of possible ways of herniated disc migration.

## Conclusion

Medial MVLs are strong connective tissue bands dividing the anterior portion of the epidural space. Lateral MVLs are more delicate and lack an annular attachment in most cases. In particular cases, MVLs could be an anatomical barrier for herniated disc migration.

## Data Availability

The original contributions presented in the study are included in the article/Supplementary Material, and further inquiries can be directed to the corresponding author/s.
